# Inhibition of the urea cycle by the environmental contaminant 2,3,7,8-tetrachlorodibenzo-*p*-dioxin increases serum ammonia levels in mice

**DOI:** 10.1016/j.jbc.2023.105500

**Published:** 2023-11-25

**Authors:** Giovan N. Cholico, Russell R. Fling, Warren J. Sink, Rance Nault, Tim Zacharewski

**Affiliations:** 1 Biochemistry and Molecular Biology, Michigan State University, East Lansing, Michigan, USA; 2 Institute for Integrative Toxicology, Michigan State University, East Lansing, Michigan, USA; 3 Microbiology & Molecular Genetics, Michigan State University, East Lansing, Michigan, USA

**Keywords:** 2,3,7,8-tetrachlorodibenzo-*p*-dioxin (TCDD), aryl hydrocarbon receptor (AHR), liver, toxicogenomics, ammonia

## Abstract

The aryl hydrocarbon receptor is a ligand-activated transcription factor known for mediating the effects of 2,3,7,8-tetrachlorodibenzo-*p*-dioxin (TCDD) and related compounds. TCDD induces nonalcoholic fatty liver disease (NAFLD)-like pathologies including simple steatosis that can progress to steatohepatitis with fibrosis and bile duct proliferation in male mice. Dose-dependent progression of steatosis to steatohepatitis with fibrosis by TCDD has been associated with metabolic reprogramming, including the disruption of amino acid metabolism. Here, we used targeted metabolomic analysis to reveal dose-dependent changes in the level of ten serum and eleven hepatic amino acids in mice upon treatment with TCDD. Bulk RNA-seq and protein analysis showed TCDD repressed CPS1, OTS, ASS1, ASL, and GLUL, all of which are associated with the urea cycle and glutamine biosynthesis. Urea and glutamine are end products of the detoxification and excretion of ammonia, a toxic byproduct of amino acid catabolism. Furthermore, we found that the catalytic activity of OTC, a rate-limiting step in the urea cycle was also dose dependently repressed. These results are consistent with an increase in circulating ammonia. Collectively, the repression of the urea and glutamate-glutamine cycles increased circulating ammonia levels and the toxicity of TCDD.

The aryl hydrocarbon receptor (AHR) is a cytosolic basic helix-loop-helix transcriptional factor that is activated following the binding of structurally diverse compounds, including environmental contaminants such as coplanar polychlorinated dioxins, dibenzofurans, and biphenyls (1). The canonical mechanism involves translocation of the liganded AHR into the nucleus where it heterodimerizes with the AHR nuclear transporter, enabling binding to dioxin response elements (DREs) and the recruitment of coregulators to elicit differential gene expression. In addition, studies show AHR interactions with other proteins including Kruppel-like factor 6 (2), estrogen receptor (3), and retinoic acid receptor (4) to regulate gene expression, following binding to nonconsensus DREs. 2,3,7,8-Tetrachlorodibenzo-*p*-dioxin (TCDD) is a persistent environmental contaminant and prototypical ligand that induces systemic toxicity, including hepatotoxicity (5, 6), while the knockout of AHR results in no pathologies across tissues (7).

Nonalcoholic fatty liver disease (NAFLD) is a metabolic disorder that affects a plethora of pathways ranging from carbohydrate metabolism, fatty acid metabolism, and amino acid metabolism (8). Many of these metabolic pathways are also dysregulated by TCDD, eliciting pathologies reminiscent of NAFLD. This includes the progression of simple steatosis to steatohepatitis with fibrosis and bile duct proliferation (9, 10) along with the disruption of lipid metabolism in male mice (11, 12).

Amino acids represent the largest pool of nitrogenous compounds and constitute the main source of organic nitrogen for the biosynthesis of other nitrogen-containing macromolecules such as proteins, hormones, neurotransmitters, heme, glutathione, and nucleotides (13). Consequently, amino acid metabolism is tightly regulated and highly coordinated with other biochemical pathways. Excess amino acids undergo catabolism, producing ammonia which is detoxified either through amidation of glutamate to yield glutamine (muscle, brain, and liver) or converted into urea in the urea cycle (liver), followed by urinary excretion (14, 15). Disruption of the urea cycle has been reported in NAFLD patients and animal models. Specifically, patients with NASH and fibrosis have lower hepatic carbamoyl-phosphate synthase 1 (CPS1) and ornithine transcarbamylase (OTC) mRNA levels and reduced CPS1 protein levels than healthy controls leading to increased circulating ammonia (16). Ammonia accumulation is also reported to promote NAFLD progression due to the activation of hepatic stellate cells (17), the liver cells primarily responsible for extracellular matrix remodeling, such as collagen deposition during fibrosis.

In this study, free amino acid levels in mice treated with TCDD were assessed using LC-MS/MS. The levels of eleven amino acids were changed in total liver extracts, while another ten were changed in serum with a concurrent elevation of serum ammonia levels. Bulk RNA-seq, as well as protein analyses, identified changes in hepatic gene expression, and protein levels associated with ammonia detoxification, specifically in the urea cycle and glutamine metabolism pathway. This included dose-dependent repression of the urea cycle enzymes CPS1, OTC, argininosuccinate synthase 1 (ASS1), and argininosuccinate lyase (ASL), as well as the glutamine-glutamate recycling enzymes glutaminase 2 (GLS2), GLUL, and glutamate dehydrogenase (GLUD). The activity of the rate-limiting urea cycle enzyme OTC was also dose dependently repressed. Collectively, the results suggest TCDD dose dependently disrupted the urea cycle and glutamine-glutamate recycling, resulting in the accumulation of ammonia in serum.

## Results

### TCDD disrupts amino acid levels

**Figure 1:**
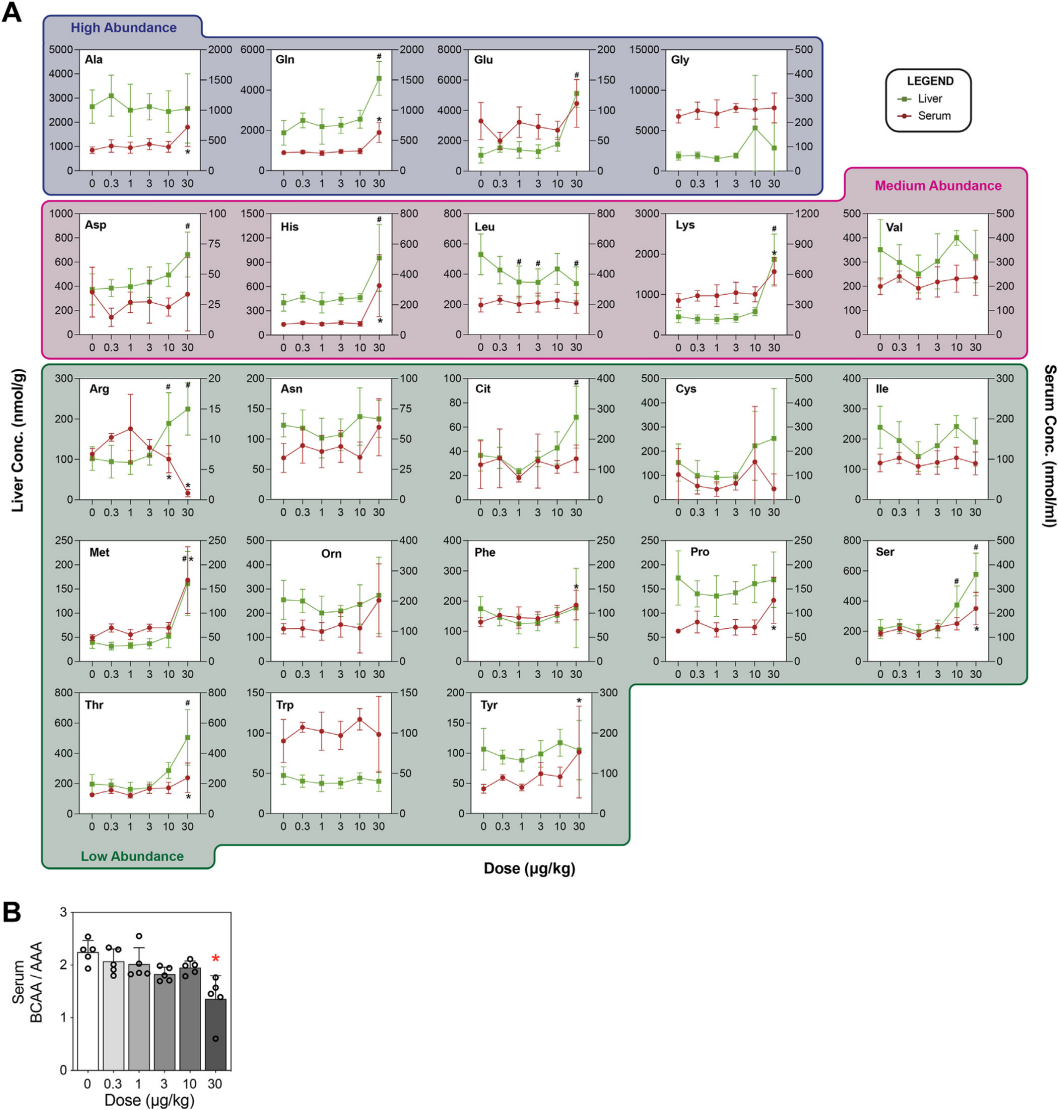
**Effect of TCDD on free amino acids.***A*, dose-dependent effect of TCDD on circulating and hepatic amino acid levels. Free amino acid levels in serum (*red*; n = 5, in some samples there was no detection) and liver extracts (*green*; n = 8, in some samples there was no detection) were assessed using targeted LC-MS. Mice were gavaged every 4 days for 28 days with TCDD or vehicle. A one-way ANOVA followed by Dunnett’s post hoc analysis was conducted to assess significance (*p*-value ≤ 0.05) in serum (∗) or liver extract (#). *B*, the ratio of serum branched-chain amino acids over serum aromatic amino acids (Fisher’s ratio) indicating liver disease progression (87). Bar graphs depict mean ± SD (n = 3). Statistical significance (∗*p* ≤ 0.05) was determined using a one-way ANOVA, followed by Dunnett’s post hoc analysis. TCDD, 2,3,7,8-Tetrachlorodibenzo-*p*-dioxin.

The effect of TCDD on amino acid levels in serum and liver extracts was assessed using LC-MS/MS. (Fig. 1*A*). In mice, arginine, histidine, isoleucine, leucine, lysine, methionine, phenylalanine, threonine, tryptophan, and valine are essential amino acids (18, 19). Overall, hepatic levels of six essential (arginine, histidine, leucine, lysine, methionine, threonine), and four nonessential (aspartate, glutamine, glutamate, serine) amino acid levels as well as the level of arginine-dependent citrulline were dysregulated (Figs. 1*A* and S1). Leucine was the only amino acid that decreased in the liver following TCDD treatment. In serum, arginine, histidine, lysine, methionine, phenylalanine, and threonine, and five nonessential amino acids (alanine, glutamine, proline, serine, tyrosine) were dysregulated by TCDD (Figs. 1*A* and S1). Arginine was the only amino acid that decreased in serum following treatment. The Fisher’s ratio, a serum marker linked to liver disease based on the relative proportion of branch chain amino acid levels relative to aromatic amino acids (BCAA/AAA), decreased dose dependently (Fig. 1*B*).

### Effects of TCDD on circulating ammonia and urea

**Figure 2:**
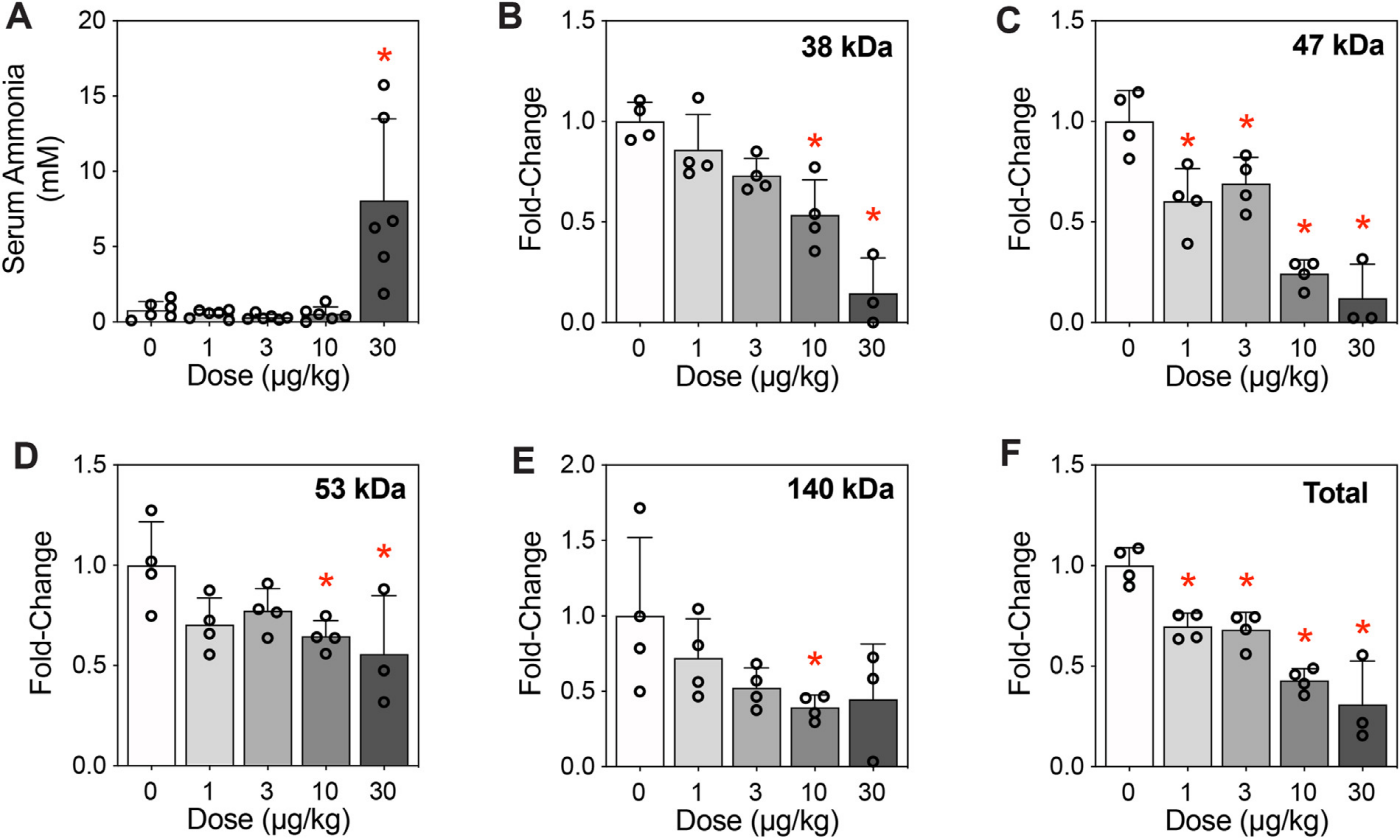
**Effect of TCDD on ammonia accumulation and protein carbamylation.***A*, ammonia levels were assessed in serum (n = 4). *B*–*E*, protein carbamylation was assessed on hepatic extracts (n = 4, 0–10 μg/kg; n = 3, 30 μg/kg) using capillary electrophoresis. Peaks corresponding to proteins of 38, 47, 53, and 140 kDa were assessed. *F*, all peaks within a sample were summed to determine total levels of carbamylated protein. Bars depict mean ± SD. Significance (∗*p* ≤ 0.05) was determined using a one-way ANOVA, followed by Dunnett’s post hoc analysis. TCDD, 2,3,7,8-Tetrachlorodibenzo-*p*-dioxin.

Dysregulation of hepatic and circulating amino acids was expected to affect ammonia levels, a toxic metabolite biotransformed and excreted as urea. Circulating ammonia levels increased in mice treated with 30 μg/kg TCDD (Fig. 2*A*). Urea can also be used as a substrate to post-translationally modify proteins by spontaneously adducting lysine residues, forming stable carbamylated derivatives (20). Carbamylation has significant implications for protein structure, function, and location, potentially impacting cellular processes. Protein carbamylation was found to dose dependently decrease for various unidentified proteins at 38, 47, 53, and 140 kDa (Fig. 2, *B*–*E*), with total carbamylated proteins showing a dose-dependent decrease consistent with the repression of the urea cycle (Fig. 2*F*). Collectively, TCDD induced the accumulation of ammonia while decreasing protein carbamylation.

### Effects of TCDD on the urea cycle

**Figure 3:**
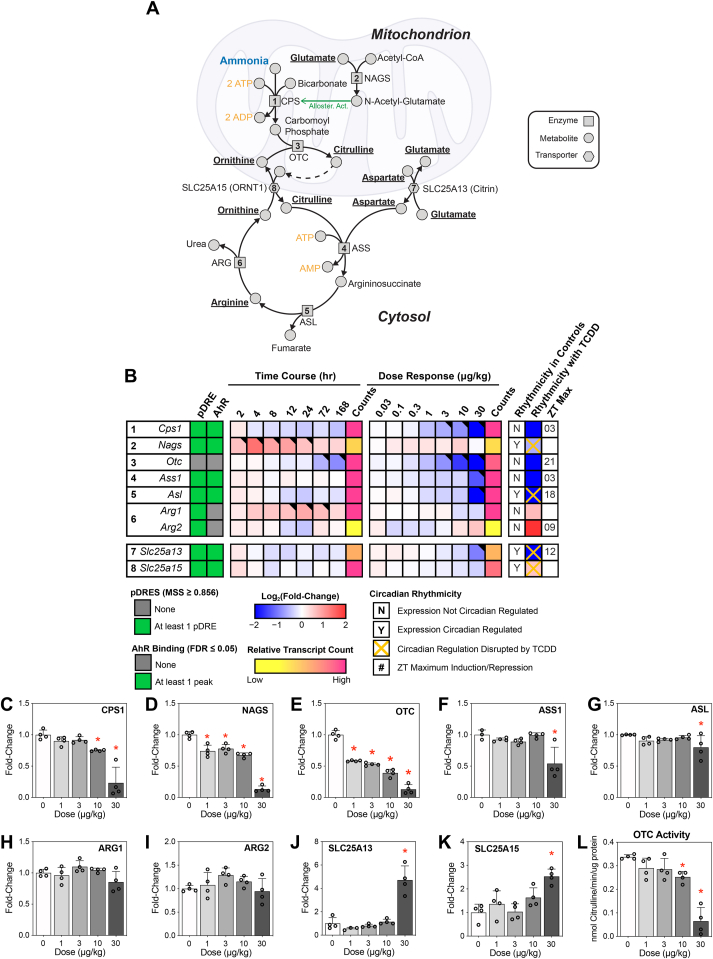
**Effect of TCDD on the urea cycle.***A*, excess ammonia is detoxified by the urea cycle. *B*, differential expression for urea cycle–related hepatic genes assessed using bulk RNA-seq. Genes are listed according to official mouse genomics informatics symbols. The presence of putative dioxin response elements (pDRE) within the gene loci and AHR enrichment at 2 h, following TCDD exposure is denoted in green. Time-dependent gene expression was assessed following a single dose of 30 μg/kg TCDD. Dose-dependent gene expression was conducted for mice (n = 3) treated with TCDD, or vehicle, every 4 days for 28 days. Circadian regulation of gene expression is denoted with a “Y,” while disruption of circadian regulation following exposure to 30 μg/kg TCDD every 4 days for 28 days is denoted with an orange “X.” The ZT with statistical significance (P1(t) > 0.8) and maximum gene induction/repression is denoted for each gene. Counts for time course and dose-response data denote the maximal number of raw read counts for each gene across all libraries. Low counts (<500) are denoted in *yellow*, while high counts (>10,000) are denoted in *pink*. Differential expression (P1(t) > 0.80) is indicated with a *black triangle* in the *top right* tile corner. Capillary electrophoresis was used to assess protein levels for (*C*) NAGS, (*D*) CPS1, (*E*) OTC, (*F*) ASS1, (*G*) ASL, (*H*) ARG1, (*I*) ARG2, (*J*) SLC25A13, (*K*) SLC25A15 from total liver extracts (n = 4). *L*, OTC activity was measured by assessing citrulline production mixed with 2,3-butanedione monoxime, and absorbance measured at 490 nm as previously described (17). Bar graphs depict mean ± SD (n = 4). Statistical significance (∗*p* ≤ 0.05) was determined using a one-way ANOVA, followed by Dunnett’s post hoc analysis. ASL, argininosuccinate lyase; ASS1, argininosuccinate synthase 1; CPS, carbamoyl-phosphate synthase; GLS2, glutaminase 2; OTC, carbamoyl-phosphate synthase 1; NAGS, N-acetyl-glutamate synthase; TCDD, 2,3,7,8-Tetrachlorodibenzo-*p*-dioxin.

TCDD-elicited disruption of the urea cycle was suggested by alterations in amino acid levels and the repression of associated gene expression involved in amino acid metabolism. Specifically, arginine, citrulline, and aspartate, all urea cycle intermediates, increased dose dependently in liver extracts, while arginine levels decreased in serum (Fig. 1, *A*). The urea cycle (Fig. 3*A*) begins with the rate-limiting carbamoyl phosphatase synthetase step (*Cps1*; dose dependently repressed 7.9-fold), which conjugates ammonia with bicarbonate to produce carbamoyl-phosphate at the expense of two ATP molecules (Fig. 3*B*). Repression of *Cps1* by TCDD was confirmed at the protein level (Fig. 3*C*). CPS1 is highly regulated and sensitive to changes in not only substrate concentrations but also allosteric activators such as N-acetyl-glutamate (21). The required allosteric activator N-acetyl-glutamate (22) is produced by the conjugation of glutamate and acetyl-CoA catalyzed by N-acetyl-glutamate synthase (*Nags*). Although TCDD had no effect on *Nags* expression there was a dose-dependent decrease in NAGS protein levels (Fig. 3*D*). The cycle continues with OTC (dose dependently repressed 57.9-fold) which conjugates carbamoyl phosphate and ornithine to form citrulline. In concordance with gene expression data, OTC protein levels were dose dependently decreased at doses as low as 1 μg/kg TCDD (Fig. 3*E*). Citrulline and aspartate are then conjugated *via Ass1* (repressed 4.6-fold) to form argininosuccinate, with ASS1 protein levels showing only a decrease at 30 μg/kg TCDD (Fig. 3*F*). *Asl* (repressed 9.5-fold) cleaves argininosuccinate into fumarate that can feed into the tricarboxylic acid (TCA) cycle, and arginine which continues in the urea cycle. ASL protein levels showed a modest decrease at 30 μg/kg TCDD (Fig. 3*G*). In the final step of the urea cycle, arginase (*Arg1*; no gene expression change), cleaves arginine into the waste product urea, and ornithine, the substrate for OTC. Protein levels for both ARG1 and ARG2, the liver- and kidney-type arginases, respectively, showed no treatment-related effects (Fig. 3, *H* and *I*). The mitochondrial transporter citrin (*Slc25a13*), which shuttles aspartate/glutamate, was repressed 4.2-fold while ORNT1 (*Slc25a15*) the citrulline/ornithine shuttle was not affected by TCDD, yet protein levels of both transporters increased at 30 μg/kg TCDD (Fig. 3, *J* and *K*). OTC enzymatic activity, a surrogate marker for urea cycle efficiency (17), was dose dependently decreased by TCDD (Fig. 3*L*). Collectively, expression of five of the eight genes associated with the urea cycle was dose dependently repressed by TCDD, with the levels of five proteins also decreasing, and two mitochondrial transporters increasing following treatment. Interestingly, CPS1 and ASS1 require ATP (Fig. 3*A*), while NAGS requires acetyl-CoA. Previous studies report that TCDD elicits an energy crisis by disrupting available ATP stores (23) as well as acetyl-CoA stores (24). In total, *Cps1*, *Nags*, *Ass1*, *Asl*, *Slc25a13*, and *Slc25a15* exhibited putative DREs (pDREs) and AHR binding.

### Effects of TCDD on the glutamine-glutamate cycle

**Figure 4:**
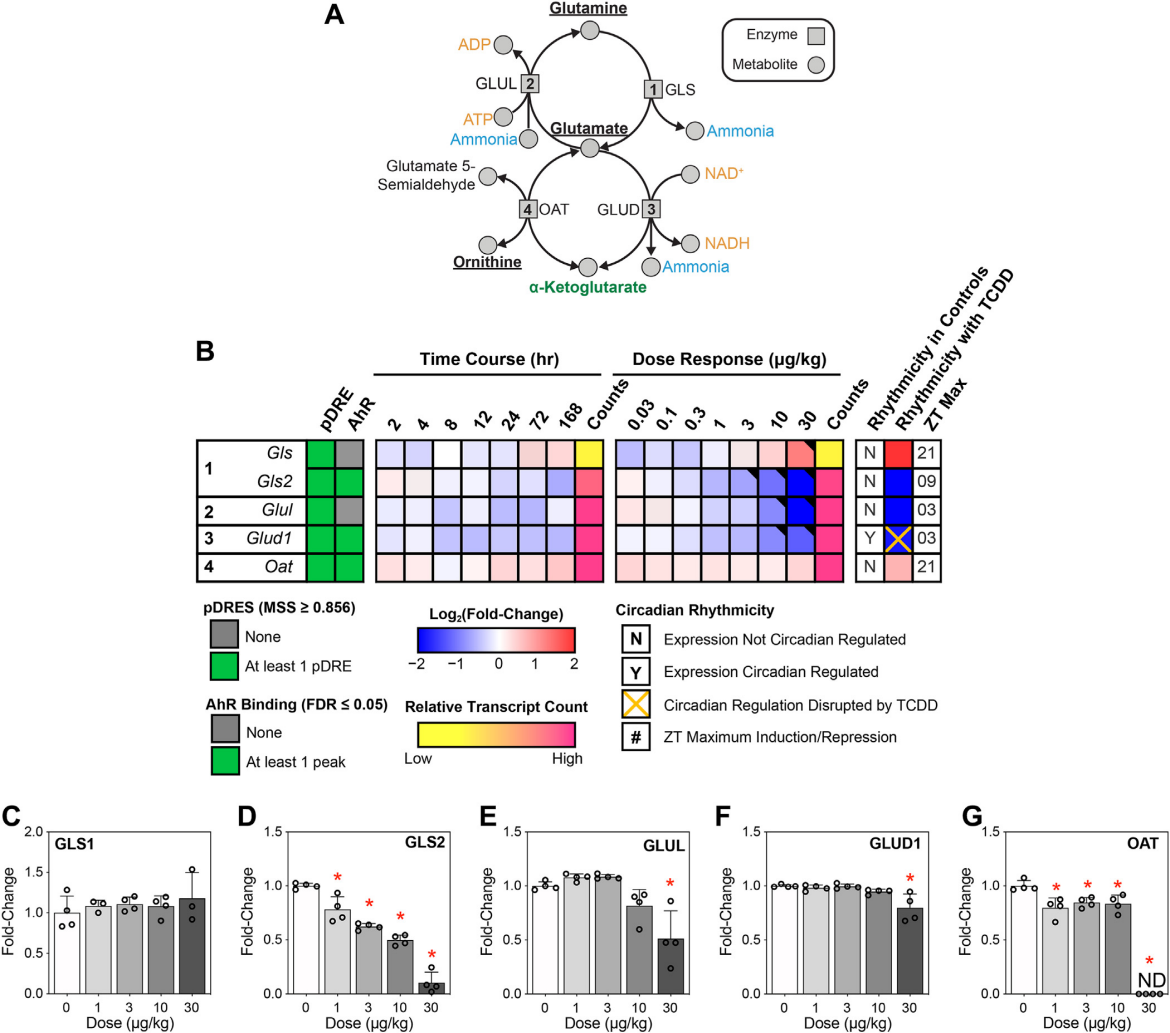
**Effect of TCDD on glutamine-glutamate cycle and ammonia homeostasis.***A*, ammonia levels are maintained through sequestration *via* glutamate and release from glutamine. *B*, differential expression in the liver for ammonia homeostasis-related genes was conducted using bulk RNA-seq. Genes are listed according to official mouse genomics informatics symbols. The presence of putative dioxin response elements (pDREs containing 5ʹ-GCGTG-3ʹ core sequence) in the gene loci, and AHR enrichment at 2 h, following TCDD exposure are denoted in *green*. Time-dependent gene expression was assessed, following a single dose of 30 μg/kg TCDD. Dose-dependent gene expression was conducted for mice treated with TCDD, or vehicle, every 4 days for 28 days (n = 3). Circadian regulation of gene expression is denoted with a “Y,” while disruption of circadian regulation following exposure to 30 μg/kg TCDD every 4 days for 28 days is denoted with an *orange* “X.” The ZT with statistically significant (P1(t) > 0.8) maximal gene induction/repression is denoted for each gene. Counts for time course and dose-response data denote the maximal number of raw read counts for each gene across all libraries. Low counts (<500) are denoted in *yellow*, while high counts (>10,000) are denoted in *pink*. Differential expression (P1(t) > 0.80) is indicated with a *black triangle* in the *top right* tile corner. Capillary electrophoresis was used to assess protein levels for (*C*) GLS, (*D*) GLS2, (*E*) GLUL, (*F*) GLUD1, and (*G*) OAT from total liver extracts (n = 4). Bar graphs depict mean ± SD (n = 4). Statistical significance (∗*p* ≤ 0.05) was determined using a one-way ANOVA, followed by Dunnett’s post hoc analysis. GLUD, glutamate dehydrogenase; GLS, glutamine synthetase; GLS2, glutaminase 2; OAT, ornithine aminotransferase; pDRE, putative dioxin response element; TCDD, 2,3,7,8-Tetrachlorodibenzo-*p*-dioxin.

Changes in the urea cycle occur concurrently with alterations in glutamine/glutamate/ammonia homeostasis, especially when liver function is hindered (25). Glutamine and glutamate exist in a harmonious state with ammonia disposal. Disruption is associated with several liver pathologies, including steatosis and fibrosis that affect ammonia management (26). In this study, TCDD dose dependently increased hepatic glutamine and glutamate levels (Fig. 1*A*), intermediates involved in ammonia recycling (Fig. 4*A*). Glutamine can be catabolized by glutamine synthetases (GLSs to release ammonia and glutamate. *Gls*, the kidney-type isoform, exhibited 4.3-fold induction, whereas *Gls2*, the liver-type isoform, was dose dependently repressed 43.0-fold by TCDD (Fig. 4*B*) (27). Isoform switching was confirmed in the present study, with GLS exhibiting a modest increasing trend (not significant), while GLS2 levels were dose dependently decreased (Fig. 4, *C* and *D*). Excess ammonia undergoes amination with glutamate catalyzed by glutamine synthetase (repressed 9.5-fold) to form glutamine in the perivenous region of the liver (28). GLUL protein levels were decreased at 30 μg/kg TCDD (Fig. 4*E*). In addition, *Glud1*, which catalyzes the oxidative deamination of glutamate to α-ketoglutarate and ammonia, was repressed 3.5-fold. GLUD1 protein levels were only modestly repressed by TCDD (Fig. 4*F*). Ornithine and α-ketoglutarate can also be converted into glutamate and glutamate 5-semialdehyde *via* ornithine aminotransferase (*Oat*; induced 1.5-fold). A modest decrease in OAT protein levels was observed for 1 to 10 μg/kg TCDD, while no protein was detected at 30 μg/kg TCDD (Fig. 4*G*). Overall, *Gls2*, *Glud1*, and *Oat* exhibited pDREs and AHR binding. These results suggest that ammonia recycling *via* the glutamine-glutamate cycle was inhibited by TCDD.

## Discussion

TCDD dose dependently altered the metabolism and levels of ten amino acids in serum and hepatic extracts. Changes in circulating amino acid levels have been associated with various liver conditions. For example, changes in branch-chain amino acids were reported in liver cirrhosis patients (29). Alterations in branch chain amino acid levels relative to aromatic amino acids (Fischer’s ratio) are associated with compromised liver function (30, 31), while elevated phenylalanine and tyrosine levels indicate advanced liver disease (32). Collectively, these changes suggest TCDD compromised liver function that likely contributed to hepatotoxicity over time despite modest increases in alanine transaminase and the absence of gross histopathological observations (10, 24).

TCDD is reported to reduce hepatic cellular energy levels due to disruption of carbohydrate metabolism and the repression of β-oxidation (11, 33). Although amino acids can serve as an alternative energy source, in most cases, their hepatic levels increased in the present study. Accordingly, most genes associated with amino acid catabolism were repressed whereas serum ammonia levels increased. Accumulating ammonia levels have deleterious effects on many organs including the brain, skeletal muscle, kidneys, lungs, and liver (14). Hyperammonemia can alter cellular function by disrupting calcium signaling (34, 35, 36), the TCA cycle (37), nitric oxide synthesis (38, 39, 40), oxidative stress (41, 42), inflammatory signaling (43), and mitochondrial dysfunction (44). Elevated ammonia also exacerbates the energy crisis burden by inhibiting anaplerosis, thereby compromising the TCA cycle (37). Under hyperammonemia conditions, muscle and brain cells utilize the glutamine-glutamate cycle to convert ammonia and glutamate into glutamine. In the present study, TCDD increased both glutamine and glutamate levels, potentially derived from peripheral detoxification in muscle and brain, which were not assessed in this study. To compensate for glutamate depletion, α-ketoglutarate is transaminated to support ammonia detoxification (45). Hyperammonemia pathologies have been reported such as the increase of intracellular calcium in various cell types such as INS-1 (rat insulinoma) (46), rat basal B cells (47), and N2a (mouse neuroblastoma) cells (48). TCDD is also reported to impair the TCA cycle (49), induce oxidative stress in various models (50, 51, 52, 53, 54), and increase inflammation in various tissues (10, 55, 56). Collectively, these results suggest that hyperammonemia may be contributing to the toxicity of TCDD and related compounds.

Under homeostatic conditions, most circulating ammonia is generated in the intestines (57, 58). Specifically, bacterial-derived urease (cleaves urea into ammonia and carbon dioxide) (59) and deaminase (60) in the large intestine, and glutaminase activity in the small intestine (61, 62) contribute to ammonia formation that accumulates in the portal circulation at levels ∼3 times higher than arterial blood (58). TCDD has been shown to disrupt the homeostasis of gut microbiota and metabolism in mice (63, 64, 65, 66). Sustained hyperammonemia may be a direct consequence of low circulating acetyl-CoA levels, which is required for the formation of N-acetyl-glutamate, an essential urea cycle cofactor that allosterically regulates CPS1. To a lesser extent, ammonia is also derived from glutamate dehydrogenase in the liver, kidneys, brain, muscle, and pancreas (67, 68). Therefore, changes in the gut flora may also explain, at least in part, TCDD-induced hyperammonemia, although further studies are needed to examine the effect on specific ammonia-producing bacterial species. Moreover, TCDD induces weight loss in murine models, characterized by muscle atrophy (69). Elevated ammonia levels have been associated with atrophy, as muscle catabolizes branched-chain amino acids to glutamate to detoxify ammonia following conversion to glutamine (68). Further studies are needed to determine if TCDD-induced reductions in muscle mass contributed to increased circulating ammonia levels, possibly creating a feedback loop resulting in further ammonia accumulation.

The conjugation of ammonia with glutamate by GLS is a major detoxification mechanism. GLS deletion in mouse liver doubles the level of circulating ammonia, indicating that hepatic glutamine synthesis plays an integral role in ammonia detoxification (70, 71). In mammals, ammonia is also biotransformed in the urea cycle to urea, which is excreted in urine. The ATP-dependent conjugation of ammonia with bicarbonate catalyzed by the mitochondrial enzyme CPS to produce carbamoyl phosphate is a rate-limiting step in the urea cycle. Carbamoyl phosphate can also be generated by carbamoyl-phosphate synthetase 2, aspartate transcarbamylase, and dihydroorotase, a trifunctional protein involved in pyrimidine synthesis but not evaluated. Interestingly, isoform switching between CPS1 and CPSII (*via* carbamoyl-phosphate synthetase 2, aspartate transcarbamylase, and dihydroorotase) occurs in hepatocellular carcinoma (HCC) to support pyrimidine synthesis and cell proliferation (72). Similarly, TCDD, a known hepatocarcinogen in rodents, induces PKM isoform switching (33), a biochemical phenomenon linked to the Warburg effect in cancerous cells as a mechanism to anabolic processes and cell division (73).

Urea cycle disorders (UCDs) are characterized by a deficiency in either i) a urea cycle enzyme, ii) a mitochondrial amino acid transporter, or iii) an N-acetyl-glutamate–producing enzyme (22), and present with hyperammonemia. In the present study, transcriptional and translational repression of CPS1, OTC, ASS1, and ASL by TCDD is consistent with elevated circulating ammonia levels. Despite the dose-dependent repression of ARG1 by TCDD, serum arginine levels decreased concurrent with elevated hepatic arginine levels. Deficiencies in the two mitochondrial amino acid transporters SLC25A15 (aka ORNT1; transports ornithine/citrulline) and SLC25A13 (aka citrin; transports glutamate/aspartate) are also reported to cause UCDs (22). However, both SLC25A15 and SLC25A13 were induced by treatment, perhaps to compensate for the diminished urea cycle activity. N-acetyl-glutamate, which is produced by NAGS, is required for CPS1 functionality. Although NAGS was not transcriptionally repressed, protein levels were dose dependently reduced. Overall, six of eight factors associated with UCDs were dose dependently repressed by TCDD, consistent with disruption resulting in the accumulation of ammonia.

TCDD dose dependently repressed CPS1, NAGS, and OTC. Mitochondrial CPS1 accounts for ∼20% of mitochondrial hepatocyte protein. Its deletion is clinically indistinguishable from N-acetyl-glutamate deficiency since CPS1 activity requires N-acetyl-glutamate as a cofactor (74, 75). Although typically identified in neonates, late-onset diagnoses are possible in infants that cannot detoxify protein catabolites due to stress, infection, or exposure to certain exogenous compounds such as valproic acid (75). CPS1 deficiencies all manifest with serum ammonia and glutamine increases and no change in citrulline levels, comparable to the changes reported in the present study (75). CPS1 also forms a nuclear complex with AHR/Kruppel-like factor 6, despite normally being in the mitochondria to support the urea cycle (76). Furthermore, it has been shown that CPS1 is required for carbamylation of some nuclear proteins (76). Therefore, the dose-dependent decrease of CPS1, leading to lower circulating carbamoyl-phosphate levels, would reduce carbamylated protein levels reported in this study. Moreover, circulating CPS1 is a marker of acute liver failure in patients with acetaminophen-induced injury with a half-life in mice sera of ∼126 min (74, 77). In HCC, CPS1 repression is proposed to increase glutamine levels to support *de novo* pyrimidine biosynthesis (72). Furthermore, cell intake of arginine has been shown to occur concomitantly with the downregulation of urea cycle enzymes in HCC (78). Together, these results indicate that the disruption of the urea cycle is consistent with TCDD-elicited hepatotoxicity.

Accumulating evidence suggests that persistent metabolic reprogramming due to chronic AHR activation is a significant contributor to the toxicity of TCDD and related compounds. Previous studies have shown that persistent AHR activation by TCDD disrupts carbohydrate and lipid metabolism (11, 12, 33). This not only compromised bioenergetics but also altered intracellular messenger levels and disrupted metabolic programming, resulting in the accumulation of toxic intermediates (24, 79). In this study, the effects on gene expression and amino acid metabolism associated with the urea cycle suggest that TCDD repressed the urea cycle, resulting in ammonia accumulation that further contributes to the toxicity burden of TCDD and related compounds due to persistent AHR activation. Further studies are needed to investigate the relevance of these effects in human models, and to examine the effect of TCDD on extrahepatic amino acid metabolism, as well as the involvement of the gut microbiome as potential sources of ammonia.

## Experimental procedures

### Animal treatment

Postnatal day 25 male C57BL/6 mice (Charles River Laboratories) were acclimated for 3 days, and then treated as previously described (11). Briefly, postnatal day 28 mice were orally gavaged with 100 μl of sesame oil or 0.03, 0.1, 0.3, 1, 3, 10, and 30 μg/kg TCDD every 4 days for 28 days. This dosing regimen, which has been used in previous studies (9, 10, 11, 12, 24, 80, 81, 82), was used to approach TCDD levels comparable to reported human serum levels, following intentional poisoning, exposure due to industrial accidents, and accumulation due to background environmental exposures (10). On day 28 of the study, mice were euthanized *via* carbon dioxide overdose, after which blood was collected by cardiac puncture. Excised liver samples were immediately flash-frozen in liquid nitrogen and stored at −80 °C. This study was conducted in accordance with relevant guidelines and regulatory procedures approved by the Michigan State University Institutional Animal Care and Use Committee (PROTO201800043) and meet the ARRIVE guidelines.

### Protein isolation, quantification, and capillary electrophoresis

Frozen liver samples (∼50 mg) were homogenized in radioimmunoprecipitation assay buffer supplemented with protease inhibitors (Sigma-Aldrich) using a Polytron PT2100 homogenizer (Kinematica). The supernatant was collected, and protein concentrations were assessed using a bicinchoninic acid assay with a bovine serum albumin standard curve. Select proteins were quantified using the WES capillary electrophoresis system (ProteinSimple), following standard manufacturer protocols. Primary antibodies and their corresponding concentrations are listed in Table S1. A secondary antirabbit detection module was used according to manufacturer protocols (ProteinSimple), except for the assessment of ARG1. For ARG1, a no secondary detection module (ProteinSimple) was used in conjunction with an antimouse secondary (ab6789; Abcam) diluted 1:750. Compass Software v6.1.0 (ProteinSimple; https://www.bio-techne.com/resources/instrument-software-download-center/compass-software-simple-western) was used to analyze chemiluminescence signal intensity. GraphPad Prism v9.5.1 (https://www.graphpad.com) was used to conduct a one-way ANOVA, followed by a Dunnett’s post hoc analysis to determine statistical significance (*p* ≤ 0.05).

### Enzymatic activity assay

Total liver homogenates were prepared as previously described to yield extracts with functional enzymes (11). Briefly, extracts were isolated from frozen liver samples using NP-40 cell lysis buffer (Thermo Fisher Scientific), supplemented with protease inhibitors, using a polytron PT2100 homogenizer (Kinematica). OTC activity was assessed as described using a standard curve of 0, 10, 20, 30, 50, 80, and 100 nmol citrulline (Thermo Fisher Scientific) in a 96-well plate (17). Enzyme activity was determined using 10 μg of total protein in a total volume of 100 μl. To each well, 25 μl of ornithine solution (50 mM), 25 μl of triethanolamine solution (2700 mM), and 25 μl of carbamyl phosphate (150 mM) were added (Thermo Fisher Scientific). A background control sample was set up in which 10 μg protein with a total volume of 100 μl was incubated with 75 μl water. The plate was sealed with aluminum sealing tape and incubated for 30 min at 37 °C. After which, 80 μl of 3:1 phosphoric acid:sulfuric acid (Thermo Fisher Scientific) were added to each well to quench the reaction, followed by 20 μl of 3% 2,3 butanedione monoxime (Thermo Fisher Scientific). The plate was then sealed and placed on a heat block of 95 °C for 30 min. After cooling to room temperature, the adhesive seal was removed, and placed on a Tecan Infinite 200 plate reader to assess absorbance at 490 nm. Enzymatic activity was calculated as nmoles of citrulline produced per min per μg of protein. GraphPad Prism v9.5.1 was used to conduct a one-way ANOVA, followed by Dunnett’s post-hoc analysis to determine statistical significance (*p* ≤ 0.05).

### Clinical chemistry

Ammonia (ab83360) were assessed in serum samples diluted 1:10, using commercially available kits (Abcam) according to the manufacturer’s specifications. GraphPad Prism v9.5.1 was used to conduct a one-way ANOVA, followed by Dunnett’s post-hoc analysis to determine statistical significance (*p* ≤ 0.05).

### Liquid chromatography–tandem mass spectrometry

A stock mixture of water: methanol (37.6: 62.4) containing isotopically labeled internal standard, cell-free amino acid mixture-^13^C, ^15^N (Sigma-Aldrich #767964), was made. To assess amino acid levels in the liver, 4.63 ml of water: methanol mixture at 4 °C was mixed with 25 mg frozen liver and homogenized for 30 s in a polytron PT2100 homogenizer (Kinematica). For serum samples, 50 μl serum was mixed with the aforementioned ratio of water: methanol. For both serum and liver samples, 2.89 ml chloroform was added and vortexed. Samples were then shaken vigorously at 4 °C for 10 min, and then centrifuged for 15 min at 5000 revolutions per minute. The aqueous layer was transferred to a new tube and dried under a steady stream of nitrogen gas. The dried pellets were resuspended in 300 μl of water and diluted in 3:1 sample stock: 4× tributylamine solution. Amino acids in serum and liver extracts were measured using a waters UPLC (Waters) attached to a Waters Quattro Micro triple quadrupole run by multiple reaction monitoring in positive ionization mode. Mobile phases and columns were previously published (83). Amino acid concentrations were calculated using analyte signal/internal standard signal and 6-point external calibration curves containing unlabeled and labeled amino acids (10 nM to 10 μM).

## Data availability

Hepatic bulk-RNAseq data sets were previously published (79, 80, 84). Time course (GSE109863), dose-response (GSE203302), and diurnal rhythmicity (GSE119780) sequencing data are available at the Gene Expression Omnibus. Genes were considered differentially expressed when the |fold-change| ≥ 1.5 and posterior probability (P1(t)) ≥ 0.8, as determined by empirical Bayes analysis (85). Diurnal gene expression rhythmicity was determined using JTK_CYCLE as previously described (80). Putative DREpDREs, https://doi.org/10.7910/DVN/JASCVZ) data were previously published (86). pDREs were considered functional with a MSS ≥ 0.856 and associated with genes when located 10 kb upstream of the transcription start site to the transcription end site. AHR chromatin binding 2 h after TCDD exposure was determined using previously published AHR chromain immuoprecipitation sequencing data (GSE97634) (86). Chromain immuoprecipitation sequencing analysis used a false discovery rate ≤ 0.05.

## Supporting information

This article contains supporting information.

## Conflict of interest

The authors declare that they have no conflicts of interest with the contents of this article.
