# Supplementary material for: Inhibition of the urea cycle by the environmental contaminant 2,3,7,8-tetrachlorodibenzo-p-dioxin increases serum ammonia levels in mice
Source: J Biol Chem. 2023 Nov 25;300(1):105500. doi: 10.1016/j.jbc.2023.105500 (PMC10731612; doi:10.1016/j.jbc.2023.105500)
Supplement: Supporting information [file mmc1.docx]

**Supporting Information**

**Inhibition of the urea cycle by the environmental contaminant**

**2,3,7,8-tetrachlorodibenzo-*p*-dioxin increases serum ammonia levels in mice**

Giovan N. Cholico^1,2^, Russell R. Fling^2,3^, Warren J. Sink^1,2^, Rance Nault^1,2^, & Tim Zacharewski^1,2,*^

^1^Biochemistry and Molecular Biology, Michigan State University, East Lansing, MI 48824, USA.

^2^Institute for Integrative Toxicology, Michigan State University, East Lansing, MI 48824, USA.

^3^Microbiology & Molecular Genetics, Michigan State University, East Lansing, MI 48824, USA.

*Correspondence

Tim Zacharewski

Michigan State University

Department of Biochemistry & Molecular Biology

Biochemistry Building

603 Wilson Road

East Lansing, MI 48824

tzachare@msu.edu

**RUNNING TITLE**

TCDD increases serum ammonia in mice

**KEYWORDS**

2,3,7,8-tetrachlorodibenzo-*p*-dioxin (TCDD); aryl hydrocarbon receptor (AHR); liver; toxicogenomics; ammonia

**Supplementary Figure 1**


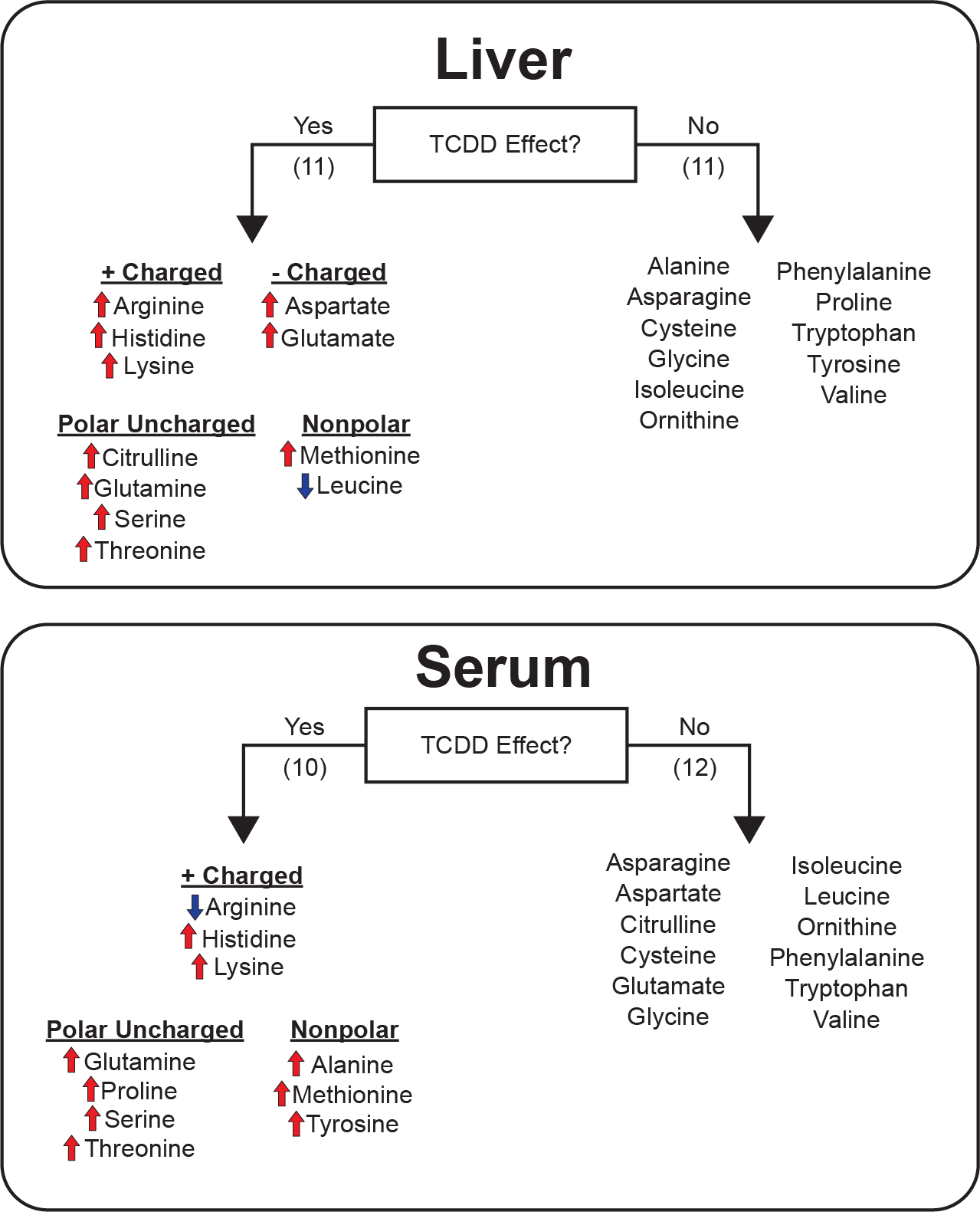


**Figure S1.** Summary of TCDD effects on amino acid levels in hepatic extracts and serum. Amino acids were assessed using targeted liquid chromatography-mass spectrometry using internal standards. Levels that increased are denoted with a red arrow while those that were repressed are denoted with a blue arrow. Induced and repressed amino acids are grouped by side-chain properties.

**Table S1**

Capillary electrophoresis was used to assess protein levels in liver extracts. The following primary antibodies at the corresponding concentrations were used.

| **Protein** | **Antibody Manufacturer** | **Catalog Number** | **Concentration** |
| --- | --- | --- | --- |
| ARG1 | Proteintech | 66129-1-Ig | 1:200 |
| ARG2 | Proteintech | 14825-1-AP | 1:200 |
| ASL | Proteintech | 16645-1-AP | 1:100 |
| ASS1 | Proteintech | 16210-1-AP | 1:200 |
| Carbamylation | Cayman | 22428 | 1:30 |
| GLS | Proteintech | 12855-1-AP | 1:50 |
| GLS2 | Abcam | ab113509 | 1:200 |
| GLUD1 | Abclonal | A7631 | 1:200 |
| GLUL | Proteintech | 11037-2-AP | 1:200 |
| NAGS | Proteintech | 21566-1-AP | 1:50 |
| OAT | Proteintech | 17089-1-AP | 1:200 |
| OTC | Proteintech | 26470-1-AP | 1:50 |
| SLC25A13 | Proteintech | 10789-1-AP | 1:50 |
| SLC25A15 | Proteintech | 15317-1-AP | 1:50 |
